# Percutaneous Ablation for Hepatocellular Carcinoma: Comparison of Various Ablation Techniques and Surgery

**DOI:** 10.1155/2018/4756147

**Published:** 2018-06-03

**Authors:** Shuichiro Shiina, Koki Sato, Ryosuke Tateishi, Motonori Shimizu, Hideko Ohama, Takeshi Hatanaka, Masashi Takawa, Hiroaki Nagamatsu, Yasuharu Imai

**Affiliations:** ^1^Department of Gastroenterology, Juntendo University School of Medicine, Japan; ^2^Department of Gastroenterology, The University of Tokyo, Japan

## Abstract

Image-guided percutaneous ablation is considered best in the treatment of early-stage hepatocellular carcinoma (HCC). Ablation is potentially curative, minimally invasive, and easily repeatable for recurrence. Ethanol injection used to be the standard in ablation. However, radiofrequency ablation has recently been the most prevailing ablation method for HCC. Many investigators have reported that radiofrequency ablation is superior to ethanol injection, from the viewpoints of treatment response, local tumor curativity, and overall survival. New-generation microwave ablation can create a larger ablation volume in a shorter time period. Further comparison studies are, however, mandatory between radiofrequency ablation and microwave ablation, especially in terms of complications and long-term survival. Irreversible electroporation, which is a non-thermal ablation method that delivers short electric pulses to induce cell death due to apoptosis, requires further studies, especially in terms of long-term outcomes. It is considerably difficult to compare outcomes in ablation with those in surgical resection. However, radiofrequency ablation seems to be a satisfactory alternative to resection for HCC 3 cm or smaller in Child-Pugh class A or B cirrhosis. Furthermore, radiofrequency ablation may be a first-line treatment in HCC 2 cm or smaller in Child-Pugh class A or B cirrhosis. Various innovations would further improve outcomes in ablation. Training programs may be effective in providing an excellent opportunity to understand basic concepts and learn cardinal skills for successful ablation. Sophisticated ablation would be more than an adequate alternative of surgery for small- and possibly middle-sized HCC.

## 1. Introduction

Hepatocellular carcinoma (HCC) is the sixth in prevalence and the second in mortality among malignant neoplasms in the world [1]. Currently, almost 80% of victims are found in Asia, and the global incidence of HCC is increasing steadily [2, 3]. Surgical resection can be applicable in only 20 % of HCC patients [4]. Furthermore, HCC frequently recurs even after apparently curative resection. Liver transplantation, which is the best therapeutic option in some patients because it can be a treatment not only for HCC but also for cirrhosis, plays a limited role by organ donor shortage. Thus, various nonsurgical therapies have developed [3, 5, 6]. Among these, image-guided percutaneous ablation is regarded as best in the treatment of early-stage HCC. It includes ethanol injection [7–9], microwave ablation (MWA) [10], radiofrequency ablation (RFA) [11–13], irreversible electroporation (IRE), and cryoablation. Ablation can be curative, minimally invasive, and easily repeatable for recurrence. Ablation is generally indicated on patients with small HCC, preferably for those with Child-Pugh class A or B liver dysfunction, up to three tumors each 3 cm or smaller in diameter [14, 15].

## 2. Ethanol Injection

Percutaneous ethanol injection was first described in the early 1980s [7–9] and had long been the standard in ablation. It is a well-tolerated, low-cost, and considerably safe treatment. Survival of patients who underwent ethanol injection has been reported to be 38–60% at 5 years [16–19]. In our study of 685 primary HCC patients on whom we performed 2,147 ethanol injection treatments, with a median follow-up of 51.6 months, survival rates were 49.0%, 17.9%, and 7.2% at 5, 10, and 20 years, respectively [19]. It has been reported that local tumor progression rates after percutaneous ethanol injection were 6–31%, which were significantly related to the size of tumor [16, 18, 20, 21]. There has been a general agreement that percutaneous ethanol injection is a safe procedure, with mortality and morbidity of 0–3.2% and 0–0.4%, respectively [18–20, 22]. Nowadays, ethanol injection is a treatment of choice only in cases in which RFA cannot be feasible because of either enterobiliary reflux, adhesion of the tumor with the gastrointestinal tract, or other reasons [15].

## 3. RFA

RFA uses high-frequency alternating current to destroy solid tumor tissue. Radiofrequency energy emitted from the exposed tip of the electrode is converted into heat. Heat is conducted considerably homogeneously in all directions; the capsule or septa of the lesion may not be a barrier of the conduction to a great degree. There are three types of electrodes: multitined expandable electrodes, internally cooled ones, and perfusion ones. RFA has recently been the most prevailing ablation technique for HCC [15]. It has been reported that survival at 5 years was 39.9–68.5% [14, 23–27]. In our study of 1,170 primary HCC patients on whom we performed 2,982 RFA treatments, with a median follow-up of 38.2 months, survival rates were 60.2 % and 27.3 % at 5 and 10 years, respectively [14]. It has been reported that local tumor progression rates after RFA were 2.4–27.0% [14, 23–27]. It has been reported that mortality and morbidity of RFA were 0.9–7.9% and 0–1.5%, respectively [14, 23–26]. Various clinical attempts, such as combination of transcatheter arterial chemoembolization followed by RFA [28] and hepatic arterial balloon occlusion during RFA [29], have been conducted to increase the ablation volume by decreasing the cooling effect of the arterial flow. There have also been some studies in which they say that multipolar RFA would be useful to increase the volume of ablation and reduce local tumor progression [30].

## 4. MWA

In MWA, tumor tissue is destroyed by direct hyperthermic injury produced by electromagnetic wave emitted from the noninsulated portions of the antenna. Microwave coagulation has been used in transaction of the liver to control bleeding from planes during resection. The first-generation MWA for clinical practice was reported in the 1990s [10]. However, its necrotic volume was small. It was prolate spheroid, 1.5 cm in short diameter and 2.5 cm in long diameter. Still worse, antenna shaft became hot from reflected power in the first-generation MWA, which results in development of pleural effusion or skin burn at the insertion site. We shifted from ethanol injection and the first-generation MWA to RFA in Japan [31].

MWA is, however, considered to have physical advantages in comparison with RFA. These advantages include a larger volume of active heating resulting in shorter procedure times, insensitivity to carbonization, higher tissue temperatures beyond the threshold of water vaporization, and less susceptibility to the heat sink effect of blood flow which results in incomplete ablation [32–34]. Thus, new-generation MWA systems incorporating water or gas antenna cooling and high-power generation have been developed and have recently been attracting large attention [35]. New-generation MWA may create a more predictable ablation zone and a larger ablation volume in a shorter time period. However, its clinical data have been insufficient compared with that of RFA. Further studies are mandatory especially in terms of long-term survival [36, 37].

## 5. IRE

IRE is a non-thermal ablation treatment that delivers short electric pulses to induce cell death due to apoptosis. The basic principle of IRE is to create irreversible pores in cellular bilipid membranes by subjecting them to a series of high voltage (>640 V/cm) and high intensity (>20 A) electrical pulses of short duration (70–100 *μ*sec) [38]. With this method, the skeleton of connective tissue, vessels, and bile ducts are largely preserved [39]. IRE seems to be an attractive alternative option for tumors near the porta hepatis or others in which thermal ablations are risky to be performed [40, 41]. However, IRE is more invasive and troublesome because general anesthesia with muscular blockade is needed. In addition, IRE also produces some degree of thermal effects which can injure bile ducts and other structures. Further studies are mandatory in IRE especially in terms of long-term outcomes.

## 6. Cryoablation

In contrast to RFA and MWA, cryoablation uses extremely low temperature to kill tumors. Tumor tissue is destroyed by both direct and indirect effects. The direct effect is a result of intra-and extracellular ice crystal formation and solute-solvent shifts, which induce cell dehydration and rupture. The indirect effect resulted from the vascular injury which would result in ischemic hypoxia. Apoptosis and immunomodulation may also play a role in cell injury [42]. Cryoablation has an advantage of its precise monitoring of ablated area during the procedure by various imaging modalities, such as CT, MRI, or ultrasound [43], therefore optimally controlling the treatment effects. A meta-analysis concluded that RFA is superior to cryoablation from the viewpoints of complications, local recurrence of patient, and local recurrence of tumor although there was no significant difference in mortality [44]. However, a randomized controlled trial said that local tumor progression is significantly less frequent in cryoablation than in RFA, although complications, tumor-free survival rates, and overall survival rates were not significantly different between the two techniques [45].

## 7. Comparison among Percutaneous Ablation Therapies

Six randomized controlled trials have been reported to compare RFA with ethanol injection. Superiority of RFA to ethanol injection, from the viewpoints of treatment response, local tumor curativity, and overall survival, has been found in four of them [13, 46–48], while the other two trials showed that the overall survival was not significantly different between them [49]. Ethanol injection, however, does not need special instruments and is inexpensive [50]. Ethanol injection may be an option in very small HCC.

Regarding the first-generation MWA, a randomized controlled trial to compare it with RFA demonstrated that the number of treatment sessions was smaller in RFA, although there was no statistically significant difference in terms of complete therapeutic effect, major complication, and local tumor progression between them [51]. A cohort study to compare the first-generation MWA with RFA for HCC of up to 2 cm in diameter showed that RFA was more effective than the first-generation MWA, because there was a significant difference in terms of treatment sessions, size of necrotic area, local recurrence rate, cumulative survival rate, adverse events of pain, fever, biliary injury, pleural effusion, and ascites between the two therapies [52].

Regarding new-generation MWA, many studies failed to show that new-generation MWA is superior to RFA from the viewpoint of local control and overall survival. A cohort study to compare it with RFA showed that there was no significant difference in complete therapeutic response, residual foci of untreated disease rate, recurrence rate, or progression-free survival between the two therapies [53]. Another cohort study to compare new-generation MWA with RFA for HCC up to 5 cm in diameter showed that there was no significant difference in complete ablation, local tumor progression, overall survival, or disease-free survival rates [54]. Another study also failed to show that there was a significant difference in terms of complete ablation, local recurrence, major adverse events, disease-free survival, or overall survival rates between the two therapies [55]. Another study to compare the two therapies for HCC within the Milan criteria did not demonstrate that complete ablation, local recurrence, disease-free survival, cumulative survival, or major complication rates were different between them, either [56]. Another comparative study showed that there was no significant difference in complete ablation, complication, de novo lesions, portal vein thrombosis, abdominal lymphadenopathy, and overall survival rates between the two therapies, while local recurrence rates were significantly lower in MWA [57]. Further comparison studies are mandatory between RFA and new-generation MWA, especially in terms of adverse events and long-term survival.

## 8. Comparison between Percutaneous Ablation Therapies and Surgical Resection

Comparison of ablation with surgical resection is considerably difficult; the indications are somewhat different between the two treatments. Patients of multiple lesions, advanced age, or more deteriorated liver function tend to undergo ablation while those of a large tumor tend to receive surgical resection. Furthermore, both treatments are highly operator-dependent and their indications may be different from institution to institution. Thus, a patient who is decided by medical professionals to undergo ablation or surgical resection at an institution may not be given the same treatment at others.

There have been four randomized controlled trials to compare RFA with surgical resection. Three of them demonstrated that overall survival was not significantly different between the two treatments. A study on patients with a solitary HCC up to 5 cm in diameter showed that overall survival and disease-free survival were not statistically different between the two treatments, but adverse events were more frequent and severe in surgical resection [58]. Another study on patients with nodular diameters of less than 4 cm and one or two nodules showed that there were no significant differences between the two treatments in overall survival and recurrence-free survival [59]. In another trial on patients with HCC up to 3 cm in diameter, there was no significant difference of the disease-free and overall survival between the two treatments, although the postoperative adverse events were significantly more frequent and hospital stay was significantly longer in surgical resection [60]. Only the remaining study on patients within the Milan criteria showed that there was a significant difference in overall survival and recurrence-free survival between the two treatments [61]. There was a randomized controlled trial to compare chemoembolization plus RFA with partial hepatectomy for HCC within the Milan criteria, which said that partial hepatectomy had better overall and recurrence-free survival than the combination of chemoembolization with RFA [61, 62]. However, it does not seem to be widely used to combine chemoembolization with RFA for HCC within the Milan criteria.

Concerning overall survival, some nonrandomized comparative studies reported that RFA had similar outcomes to resection [63–76] while others found that resection had better survival [74, 77–82]. There was a study in which RFA showed a better long-term survival than surgical resection after propensity score analysis [83]. There was another study which reported that, in HCC cases of 2 cm or smaller, major adverse events occurred significantly more often in surgical resection than in RFA and overall survival was better in RFA than in surgical resection [84]. Even in studies in which surgical resection was reported to be superior to RFA, there were no significant differences in overall survival between the two treatments in patients with HCC 2 cm or smaller [80] or 3 cm or smaller in diameter [74, 78, 79]. RFA was associated with less frequent adverse events [71, 79] and shorter hospital stay [71]. From the viewpoint of cost-effectiveness, RFA may be superior to surgical resection [85]. RFA seems to be a satisfactory alternative to resection for HCC up to 3 cm in diameter in Child-Pugh class A or B cirrhosis. Furthermore, RFA may be a first-line treatment in HCC up to 2 cm in diameter in Child-Pugh class A or B cirrhosis.

Most studies reported that recurrence-free survival was better in surgical resection than in RFA, although overall survival was not significantly different between the two treatments. This is probably because surgical resection sacrifices a much larger volume of non-cancerous liver tissue. It may remove some latent metastases and reduce new carcinogenesis, resulting in lower recurrence rates. However, it may lead to liver decompensation in some cases. In addition, most recurrence can be treated curatively by iterative RFA but not by repeated surgical resection. In our study, the first recurrence was treated by iterative RFA in 659 (88.9 %) of the 741 patients. In the remaining, transarterial chemoembolization in 69 (9.3 %), systemic chemotherapy in 4 (0.5 %), surgical resection in 3 (0.4 %), radiation therapy in 2 (0.3 %), and supportive care in 4 (0.5 %) were chosen [14]. On the other hand, repeated surgical resection can be an option in only 20-30% of patients with recurrent HCC.

## 9. Various Innovations and Sophisticated Instruments

**Figure 1:**
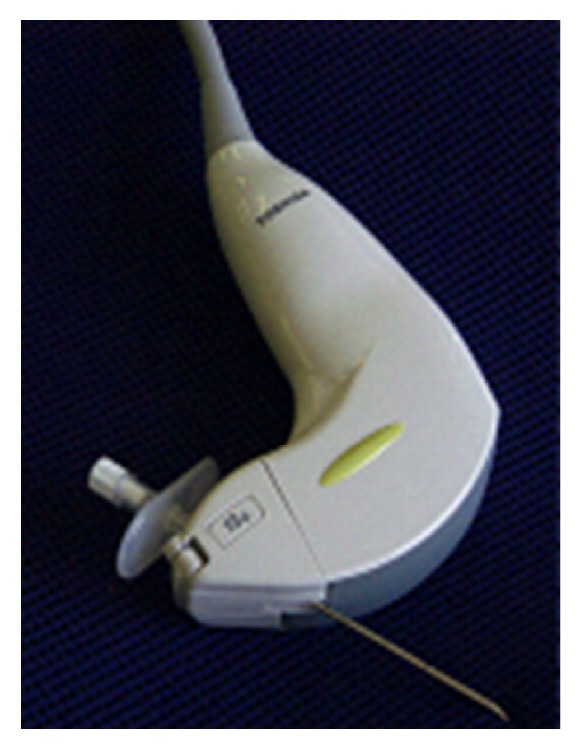
We participated in development of a dedicated ultrasonic transducer and have used it in 12,000 procedures of radiofrequency ablation. The dedicated ultrasonic transducer has the following advantages: (1) needle slot is located inside the transducer, (2) a puncture angle of 100 degrees is available in addition to 55, 70, and 85 degrees, (3) the same image is obtained as a regular convex transducer generates, (4) a puncture attachment is unified with the transducer, and (5) it is capable of multimodality fusion imaging.

**Figure 2:**
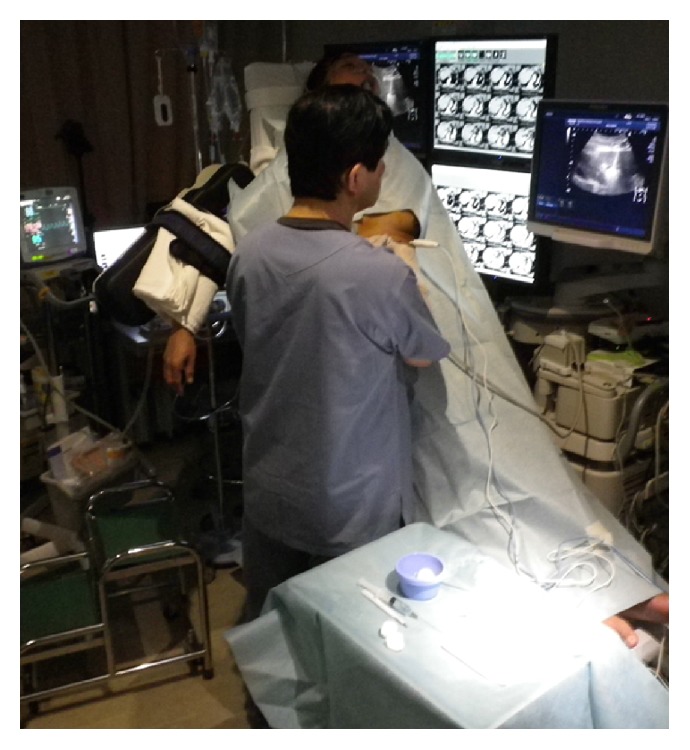
We developed a dedicated procedure bed. Using this bed, we can keep a patient in an optimal position, such as right hemilateral decubitus position, left hemilateral decubitus position, head-up position, sitting position, and almost standing position.

We developed a dedicated ultrasound transducer for puncture (PVT-350BTP, Toshiba, Japan) (Figure 1) in corporation with Toshiba. We have performed RFA over 10,000 cases of liver tumors using this dedicated ultrasound probe. Contrast-enhanced ultrasound is useful to detect viable tumor persistence following locoregional treatment (either ablation or chemoembolization). There are cases in which B-mode ultrasound cannot clearly identify a viable portion of the tumor. Using ultrasound contrast agents, vascular imaging shows a hypervascular area which represents the residual cancer tissue. We developed a dedicated procedure bed (Figure 2). Using this bed, we can keep a patient in an optimal position. Multimodality fusion imaging is also useful to detect tumors not clearly identified by ultrasound. It is a system in which fused CT or MR images created from previously acquired imaging data demonstrate the same plane and move synchronously with** real-time** ultrasound images. Various innovations would further improve outcomes in percutaneous ablation.

## 10. Training Programs

**Figure 3:**
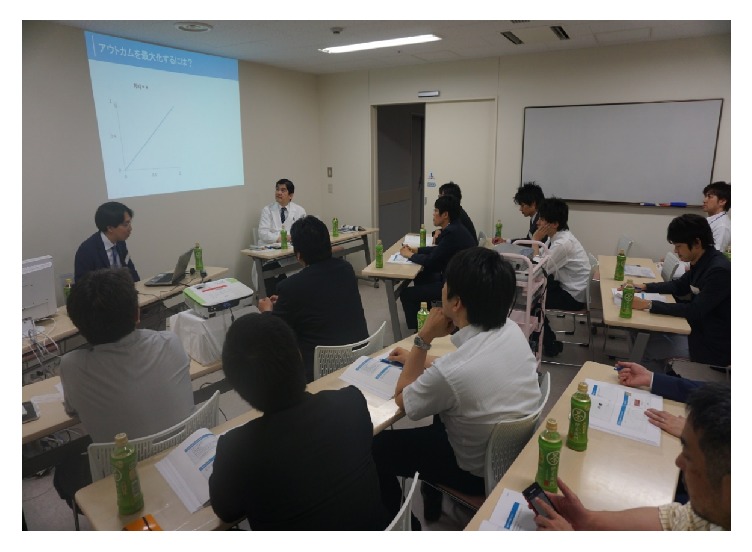
Lecture topics are current status of ablation, ablation systems, ultrasound systems, various techniques in ablation, and others.

**Figure 4:**
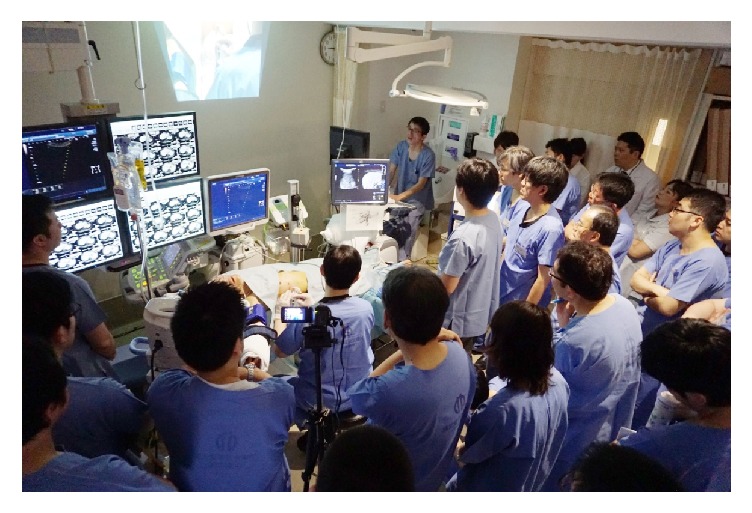
In live demonstrations, we perform ablation on various cases: a case of first diagnosed cancer not difficult to ablate judging from its size and location, a case of a tumor beneath the diaphragm requiring artificial ascites, a case of a tumor in the caudate lobe, a case of a tumor adjacent to the heart, a case of a tumor next to portal vein or hepatic vein at porta hepatis, a case of a tumor over 5 cm in diameter, a case of more than five tumors, cases of hepatic metastasis from the colorectal cancer or the gastric cancer, a case of simple nodular type HCC with extranodular growth or confluent multinodular type HCC, a case of a tumor with unclear boundaries on ultrasound which requires contrast-enhanced ultrasound to perform RFA, a case in which a tumor cannot be detected on ultrasound and requires support of fusion imaging, and others. From these cases, we demonstrate the importance of appropriate patient posture, usefulness of our original dedicated probe for interventional procedures and our RFA dedicated operation table, and the way to carry out ablation under contrast-enhanced ultrasound guidance and with multimodality fusion imaging.

**Figure 5:**
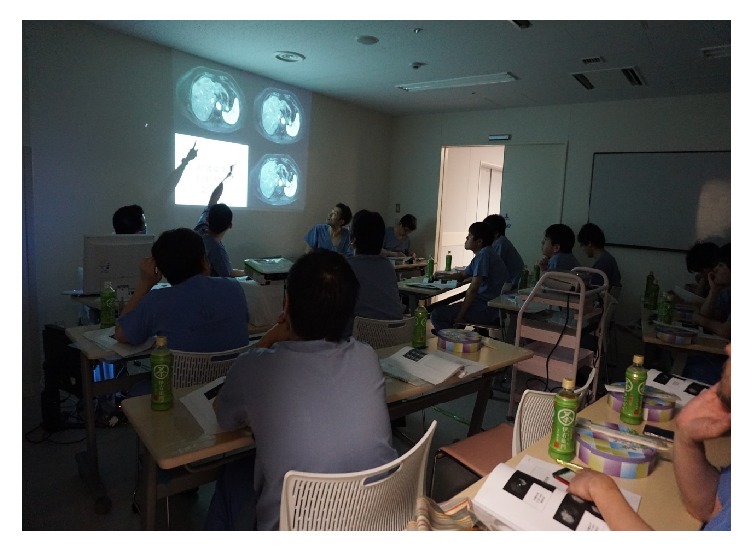
In case studies, difficult to ablate cases from participants' institutions are presented and discussed.

Because ablation appears a simple procedure, it may be done without sufficient training. However, ablation is considerably operator-dependent. Its skills and outcomes differ much from operator to operator. In order to disseminate skills and know-hows, there are some training programs for ablation. For example, our training programs are composed of comprehensive lectures (Figure 3), live demonstrations (Figure 4), and case studies (Figure 5). We have held domestic training programs 10 times, and a total of 170 doctors attended. We also have had international ones 4 times, which were successfully completed with 49 participants in total. Training programs may be effective in providing an excellent opportunity to understand basic concepts and learn cardinal skills for successful ablation.

## 11. Conclusions

Ablation has been widely performed in the treatment of HCC. Ablation is potentially curative, minimally invasive, and easily repeatable for recurrence. In RFA, outcomes in over 10-year period clearly show that RFA is a curative treatment and enables long-term survival. There are still arguments regarding whether it is proper to perform ablation on resectable cases or not. The number of patients treated by ablation, however, has been increasing. Various innovations would further improve outcomes in ablation. Training programs may be effective in acquiring necessary skills, knowledge, and experience for successful ablation. Sophisticated ablation would be more than an adequate alternative of surgery for small- and possibly middle-sized HCC.
